# Personal protective equipment for COVID‐19 among healthcare workers in an emergency department: An exploratory survey of workload, thermal discomfort and symptoms of heat strain

**DOI:** 10.1111/1742-6723.14152

**Published:** 2022-12-20

**Authors:** Andrew Hunt, Joseph Ting, Daniel Schweitzer, E‐Liisa Laakso, Ian Stewart

**Affiliations:** ^1^ School of Biomedical Sciences, Faculty of Health Queensland University of Technology Brisbane Queensland Australia; ^2^ School of Public Health and Social Work, Faculty of Health Queensland University of Technology Brisbane Queensland Australia; ^3^ Department of Emergency Medicine Mater Hospital Brisbane Queensland Australia; ^4^ Centre for Neurosciences Mater Hospital Brisbane Queensland Australia; ^5^ Wesley Hospital Brisbane Queensland Australia; ^6^ School of Medicine The University of Queensland Brisbane Queensland Australia; ^7^ Mater Research Institute The University of Queensland Brisbane Queensland Australia; ^8^ Menzies Health Institute Queensland Griffith University Gold Coast Queensland Australia; ^9^ School of Exercise and Nutrition Sciences, Faculty of Health Queensland University of Technology Brisbane Queensland Australia

**Keywords:** COVID‐19, environmental symptoms questionnaire, NASA‐TLX, PPE, subjective heat illness

## Abstract

**Objectives:**

To examine workload, thermal discomfort and heat‐related symptoms among healthcare workers (HCWs) in an Australian ED during the COVID‐19 pandemic.

**Methods:**

A cross‐sectional study design was employed among HCWs in an ED at a metropolitan hospital in Brisbane, Australia. Respondents provided demographic information including their self‐reported age, sex, height, weight, role (e.g. doctor, nurse), and whether they wore personal protective equipment (PPE) during their shift, rated as either Full PPE, Partial PPE, or usual uniform or scrubs. The workload of HCWs was assessed with the National Aeronautics and Space Administration's task load index (NASA‐TLX). Thermal discomfort was evaluated using scales from the International Organisation for Standardisation. Responders rated their subjective heat illness using the Environmental Symptoms Questionnaire.

**Results:**

Fifty‐nine HCWs completed the survey (27 male, 31 female, one prefer not to answer). Overall workload from the NASA‐TLX was 64.6 (interquartile range [IQR] 56.5–73.3) for doctors, 72.5 (IQR 63.3–83.3) for nurses and 66.7 (IQR 58.3–74.17) for other staff, representing moderate to high ratings. Eighty‐one percent reported thermal sensation to be slightly warm, warm, or hot, and 88% reported being uncomfortable, ranging from slightly to extremely. Ninety‐seven percent reported at least one heat‐strain symptom. More than 50% reported light‐headedness or headache and approximately 30% reported feeling dizzy, faint, or weak.

**Conclusions:**

ED HCWs experience thermal discomfort when wearing PPE. Combined with their workloads, HCWs experienced symptoms related to heat strain. Therefore, careful consideration should be given to managing heat strain among HCWs when wearing PPE in an ED.

## Graphical abstract

This cross‐sectional study surveyed healthcare workers' (HCWs) thermal discomfort, workload, and heat‐related symptoms when wearing personal protective equipment (PPE). ED HCWs experience thermal discomfort, moderate to high workloads, and symptoms related to heat strain when wearing PPE. Therefore, careful consideration should be given to managing heat strain among HCWs when wearing PPE in an ED.

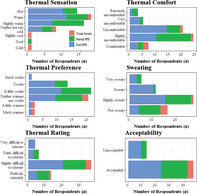

**Key findings**

- The majority of HCWs experience thermal discomfort wearing PPE.
- More than half of HCWs reported headaches, and >30% felt dizzy or faint.
- Further research into physiological strain, performance and patient outcomes is warranted.

## Introduction

Personal protective equipment (PPE) is required to protect frontline doctors, nurses and allied health professionals (healthcare workers [HCWs]) from cross‐infection. In past epidemics of highly infectious diseases such as Ebola, SARS and Zika, PPE has been critical to protecting HCWs from infection.^1^ PPE increases thermal insulation around the body and impairs evaporative heat loss.^2^, ^3^ Consequently, wearers may experience thermal discomfort and heat strain including elevated core and skin temperatures, sweat losses, and heart rates.^4^ Elevated thermal discomfort from wearing PPE may also impair the performance of complex cognitive tasks.^5^ As such, careful consideration should be given to the impacts of PPE among HCWs.

Several studies have recently reported aspects of thermal discomfort among HCWs during the COVID‐19 pandemic. Internationally, over 50% of HCWs report heat as an adverse effect of PPE.^6^, ^7^, ^8^, ^9^ Among HCWs in the UK's National Health Service, the vast majority reported feeling ‘hot’, were ‘very uncomfortable’ wearing PPE, and stated that the PPE impaired their physical performance.^5^ Almost all surveyed Dutch HCWs reported heat strain symptoms when wearing PPE compared to only 30% when not wearing PPE.^10^ In Singapore and India, over three quarters of HCWs experienced excessive sweating, thirst and exhaustion.^11^ Several limitations of these studies exist, including the use of a range of questionnaires and scales for workload, thermal discomfort, and heat‐related symptoms, with varied levels of validation and standardisation. Moreover, these studies did not focus on HCWs in EDs, nor has a study been conducted on the Australian medical population. Therefore, the present study aimed to examine workload, thermal discomfort, and heat‐related symptoms among HCWs in an Australian ED during the COVID‐19 pandemic.

## Methods

### Participants

A cross‐sectional study design was employed to assess thermal comfort, workload and symptoms of heat strain among emergency and critical care staff at a metropolitan hospital in Brisbane, Australia. All staff were eligible to complete an anonymous online survey at the end of their work shift. Links to the survey were distributed via email and made available in break rooms. Respondents provided demographic information including their self‐reported age, sex, height, weight, role (e.g. doctor, nurse), and whether they wore PPE during their shift, rated as either Full PPE (including a gown/apron, an N95 mask, eye protection and gloves), Partial PPE (some, but not all, of the items listed for full PPE), or usual uniform or scrubs. PPE requirements were defined by the Australian Government's contact and droplet precautions guidelines for PPE use in routine care of patients with suspected, probable or confirmed COVID‐19.^12^ The data collection period occurred during the COVID‐19 pandemic and spanned from June 2021 to March 2022. While the environment within the ED was air conditioned (20–24°C), Brisbane summer and winter temperatures (minimum and maximum) in Brisbane average 22–30°C and 10–22°C, respectively. Participants were informed about the survey prior to commencing, and submission of their responses constituted consent to participate. The study was approved by the Mater Misericordiae Ltd. Human Research Ethics Committee (EC00332, project ID 69541). The study is reported in accordance with STROBE guidelines.

### Standardised scales and assessments

The survey consisted of scales and indices for evaluating three aspects of heat stress: workload, thermal discomfort and heat strain symptoms. Optionally, participants were also invited to write comments about their experiences wearing PPE during their shift.

#### Workload

Workload is an important consideration for assessing heat stress, as the physical aspects of work increase metabolic heat production and non‐physical aspects may be exacerbated by heat strain. The National Aeronautics and Space Administration's task load index (NASA‐TLX) was used to assess six aspects of staff workload over the shift.^13^ As previously described by Hoonakker *et al*.^14^ with intensive care nurses, respondents were asked to rate six subscales on a 20‐step scale, resulting in a score from 0 (low) to 100 (high), including physical, mental, and temporal demands, as well as their performance, effort and frustration (Appendix S1). The raw scores were used for analysis, and overall workload (the average of the six sub‐scales) was also calculated for each participant.^14^

#### Thermal discomfort

The International Organisation for Standardisation (ISO) provides comprehensive guidance on assessing the thermal environment in various occupational settings.^15^ The guidance aims to identify concerns affecting health and safety and to outline risk management strategies according to the specific context observed. Respondents were asked to answer the questions with respect to how they felt while wearing PPE (or with respect to how they felt wearing their usual scrubs/uniform). They rated their thermal comfort on a 5‐point scale from comfortable to extremely uncomfortable, thermal sensation on a 7‐point scale from hot to cold, thermal preference on a 7‐point scale from much warmer to much cooler, stickiness (sweatiness) on a 4‐point scale from not sweaty to very sweaty, tolerability on a 5‐point scale from perfectly tolerable to intolerable, and acceptance as acceptable or unacceptable^15^ (Appendix S2).

#### Heat strain symptoms

The Environmental Symptoms Questionnaire (ESQ) contains a subscale known as the index of subjective heat illness (SHI).^16^, ^17^ The ESQ‐SHI comprises 22 symptoms related to exertional heat illness that are rated on a 6‐point scale from 0 (not at all) to 5 (extreme) (Appendix S3). The sum of each symptom's score is taken as the overall index value. The ESQ‐SHI has also been used in other populations wearing PPE including American football players,^18^ and military personnel.^19^, ^20^

### Data analysis

Continuous variables were assessed by the Shapiro–Wilk test and summarised as the median and interquartile range (IQR) when not normally distributed. Categorial variables are summarised as counts and percentages. Thermal discomfort was examined by the levels of PPE worn by staff. The NASA‐TLX subscales were compared among doctors, nurses and other staff. Associations between continuous variables were examined with Pearson correlations. Differences in group medians were assessed with the Wilcoxon rank‐sum test or Kruskal Wallis test for continuous variables, and Fisher's exact tests were used to compare categorical variables with the low sample size and cell counts. Statistical significance was accepted at *α* < 0.05. Data visualisation and analysis was performed in R Studio (version 1.3.1093) with the following packages: tidyr, dplyr, userfriendlyscience, ggplot2, grid, gridextra, car, stringr, viridis and psych.

## Results

Fifty‐nine ED staff completed the survey (27 male, 31 female, one prefer not to answer), representing a response rate of 41%. Response rates for nurses (*n* = 18) and doctors (*n* = 32) were 17% and 94%, respectively. Other staff (*n* = 9) included allied health professionals, phlebotomists, wards persons and students. Respondents were 33 years of age (IQR 27.0–40.5 years), 172 cm (IQR 165–180 cm) in height, 75 kg (IQR 63.0–89.5 kg) in weight and had a BMI of 25 kg/m^2^ (IQR 22–28 kg/m^2^). Thirty‐two (54%, 16 doctors, 12 nurses, four other) staff reported wearing full PPE during their shift, 23 (39%, 14 doctors, six nurses, three other) wore partial PPE, and four (7%, two doctors and two other staff) wore only their usual uniform or scrubs. Doctors reported wearing PPE for 60% of their shift (IQR 10–100%), nurses also report 60% (IQR 40–97.5%), and other staff only 30% (IQR 10–50%) of their shift.

### Workload

**Figure 1:**
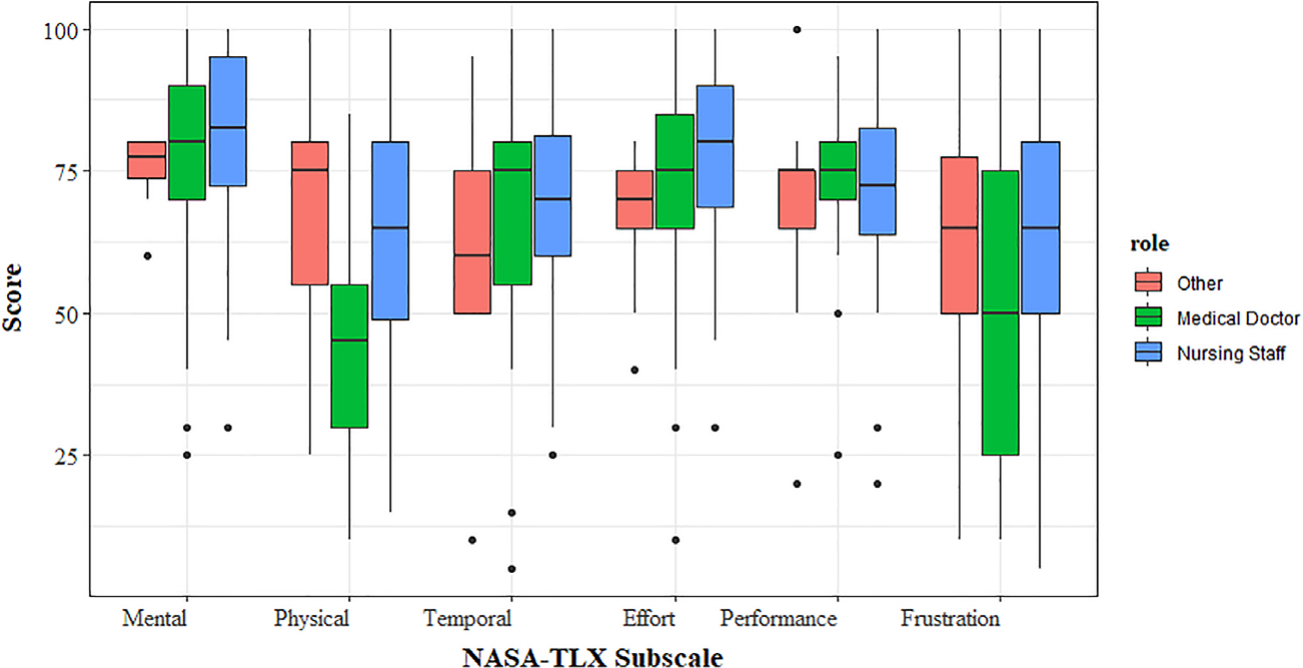
Boxplot of the National Aeronautics and Space Administration's task load index subscale scores across the staff roles.

Overall workload from the NASA‐TLX was 64.6 (IQR 56.5–73.3) for doctors, 72.5 (IQR 63.3–83.3) for nurses, and 66.7 (IQR 58.3–74.17) for other staff but was not significantly different across these roles (*H* = 3.55 (2), *P* = 0.169). Overall workload did not differ based on sex (*w* = 451.5, *P* = 0.199), nor was it related to age (*r* = 0.830, *P* = 0.543) or BMI (*r* = 0.205, *P* = 0.129). The NASA‐TLX revealed moderate to high ratings across all subscales (Fig. 1). Subscale ratings between the roles were not significantly different, with the exception of physical workload (*H*(2) = 14.97, *P* < 0.001), for which doctors tended to have lower scores than nurses and other staff.

### Thermal discomfort

**Figure 2:**
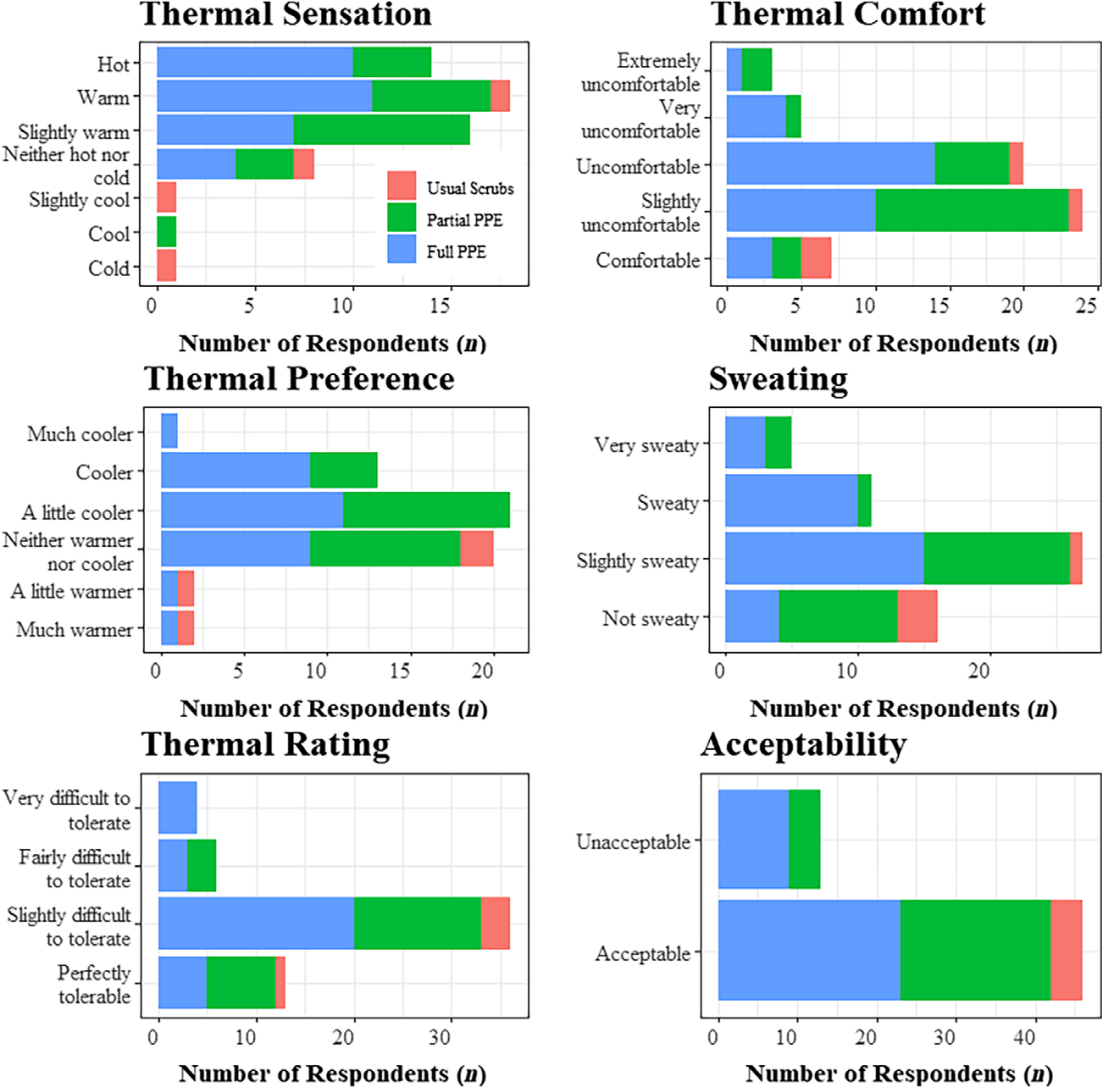
The number of respondents providing each rating of thermal discomfort across the personal protective equipment levels.

Overall, staff reported thermal discomfort during their shift (Fig. 2). Eighty‐one percent of respondents reported thermal sensation to be slightly warm, warm, or hot, and 88% reported being uncomfortable, ranging from slightly to extremely. While 34% of respondents had a preference to be neither warmer nor cooler, 58% desired to be slightly cooler or cooler. The majority (73%) of respondents reported being sweaty to some degree, mostly slightly. Although 61% described the conditions as slightly difficult to tolerate, only 22% found it unacceptable. The levels of PPE worn showed significant positive relationships with thermal sensation (*P* = 0.042) and sweatiness (*P* = 0.023).

### Heat‐related symptoms and comments

**Figure 3:**
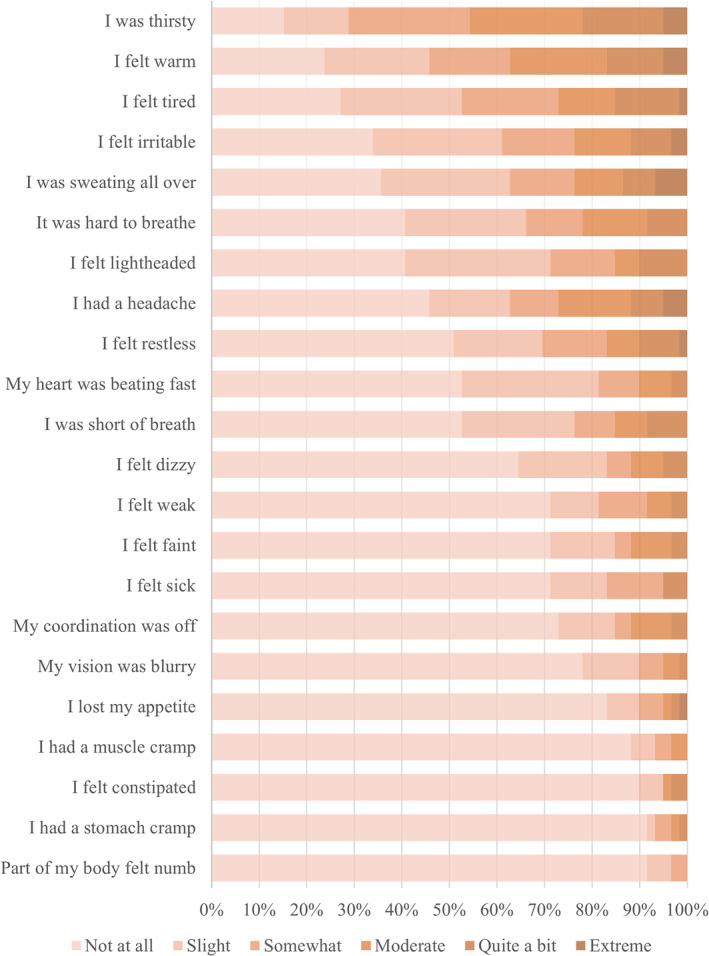
Severity ratings of heat‐related symptoms.

Staff reported a range of heat‐related symptoms during their shift (Fig. 3). Ninety‐seven percent reported experiencing at least one heat‐strain symptom. Feeling thirsty, warm, tired, and irritable were commonly reported (>60%). More than 50% reported slight or greater feelings of light‐headedness or headache and approximately 30% reported feeling dizzy, faint, or weak to a slight or greater degree. The median ESQ‐SHI was 14 (IQR 8–29). The ESQ‐SHI was not significantly correlated with age (*r* = −0.053, *P* = 0.691) or BMI (*r* = −0.040, *P* = 0.770), but was significantly positively related to overall workload (*r* = 0.366, *P* = 0.005). The ESQ‐SHI was not significantly different between roles (*H*(2) = 0.286, *P* = 0.352), PPE levels (*H*(2) = 4.107, *P* = 0.128) or sexes (*W* = 443, *P* = 0.708).

Sixteen respondents provided comments about their experience wearing PPE (Table S1). Four comments related to experience of heat, five focused on the impact of the PPE, six described how PPE affected their work practices, and one highlighted how their performance and efficiency was affected.

## Discussion

Our exploratory survey confirms that thermal discomfort among Australian HCWs in an ED is very common when wearing PPE (88%). Moreover, moderate to high workloads and heat‐related symptoms suggest HCWs are experiencing a level of heat strain. Further research into physiological strain, task performance, and patient care is warranted as effective management practices may be required to avoid excessive strain and performance deterioration.

The SARS‐CoV‐2 variant has brought about unprecedented challenges for HCWs. Importantly, a number of occupational implications have emerged, including the use of PPE across emergency and critical care settings. EDs are regularly congested, and clinicians frequently must multitask across a range of cognitively and procedurally demanding tasks. As such, there is concern that the ability to perform a range of diagnostic and procedurally demanding tasks optimally may in part be hampered by the application of PPE.^5^, ^10^ In the present study, the NASA‐TLX demonstrated overall workload among doctors, nurses, and other staff was moderate to high, similar to previous reports among intensive care nurses.^14^ Physical and temporal demands add to the thermal burden experienced by HCWs by increasing metabolic heat production, and similar to the physical demands indicated via the NASA‐TLX subscale in the present study, a previous study also confirmed moderate to vigorous physical activity among emergency nurses in Australia.^21^ Mental demands, effort and frustration, which were rated moderate to high (Fig. 1), may be exacerbated by thermal discomfort and heat strain. In addition, many individual comments related to work practices (Table S1), such as ensuring regular breaks to reduce physical demands and allow for rehydration. For these reasons, and similar to HCWs in the NHS,^5^ carefully managing HCW workloads may be an important avenue to mitigate the adverse effects of PPE.

Heat is internationally recognised as a prominent adverse effect for HCWs wearing PPE.^6^, ^7^, ^8^, ^9^ While previous research has used a range of scales and questionnaires, we followed the guidance of the ISO to evaluate thermal discomfort.^15^ Elevated thermal discomfort is concerning as HCWs have reported adverse effects of wearing PPE on the performance of complex cognitive tasks, impairing their attentional focus, decision making and problem solving abilities.^5^ Furthermore, thermal discomfort may degrade infection control because of frequent donning and doffing of PPE, reported among 77% of HCWs in the NHS.^5^ Finally, thermal discomfort may be a precursor to heat strain symptoms.

Heat strain symptoms are an indication that the physiological mechanisms regulating body temperature may be compromised. The high proportion of Australian HCWs experiencing heat strain symptoms while wearing PPE in the present study is comparable to similar populations abroad.^5^, ^10^, ^11^ Concerningly, more than 50% reported light‐headedness or headache and approximately 30% reported feeling dizzy, faint, or weak in the present study. These symptoms may predispose HCWs to accidents and injuries. Furthermore, the median ESQ‐SHI in the present study (14.0) was comparable to military personnel successfully completing an outdoor march (mean 15.0), but lower than experienced by personnel who withdraw from the march because of their symptoms (mean 28.4).^20^ This comparison highlights that wearing PPE, even in an air‐conditioned hospital environment, elevates heat strain comparably to physically demanding outdoor work without PPE. While the present study captures heat‐strain symptoms over a single shift, research among outdoor workers in Northern Australia highlights that workers experience a range of moderate to severe heat strain symptoms on daily and weekly bases.^22^ Not only do they report the adverse effects of heat on work tasks, but also on their sleep, appetite and relationships with friends and family.^22^ Concerningly, regular exposure to heat‐strain symptoms from wearing PPE may have adverse effects beyond the workplace.

Not only does the primary focus on reducing contagion risk for HCWs neglect the physiological and cognitive stresses incurred by PPE, but a range of other health concerns also arise. Skin injuries, predominantly facial, are known to occur because of device‐related pressure, prolonged moisture retention and skin tears.^23^ Furthermore, many HCWs develop PPE‐associated headaches or exacerbation of their pre‐existing headache disorders,^24^ and there are concerns about procedural dexterity and surgical performance being impaired by PPE.^25^ Overall, a comprehensive approach should be considered to implement effective management practices that mitigate excessive strain and performance deterioration while ensuring the protection offered by PPE to HCWs.

Several limitations of the present study should be acknowledged. First, the study recruited only a small sample size from a single institution, which may limit the generalisability of the findings. Of note, only four respondents wore usual scrubs (no PPE), thus caution should be exercised when interpreting the relationship found between PPE level and thermal discomfort parameters. Second, the self‐report nature of the assessments cannot rule out potential biases from respondents. Third, the cross‐sectional design of the study does not enable conclusions to be drawn regarding whether wearing PPE affects task performance and patient outcomes by elevating heat strain in HCWs. However, the level of discomfort and heat‐related symptoms reported, coupled with the individual comments suggests a potential impact is concerningly plausible. How and if PPE impacts patient care will need to be explored further through controlled research studies. Such research should objectively measure physiological responses (core and skin temperatures, heart rate and sweat loss) and indicators of task performance alongside perceptual measures when wearing standard scrubs with and without PPE.

In conclusion, ED HCWs experience thermal discomfort when wearing PPE. Combined with their workloads, HCWs experienced symptoms related to heat strain. Therefore, careful consideration should be given to managing heat strain among HCWs when wearing PPE in an ED. Further research into the effects of PPE on performance and patient outcomes is warranted.

## Supporting information


**Appendix S1.** NASA task load index.

**Appendix S2.** Thermal discomfort.

**Appendix S3.** Environmental Symptoms Questionnaire – subjective heat illness.

**Table S1.** Individual comments about wearing PPE.

## Data Availability

The data that support the findings of this study are available from the corresponding author upon reasonable request.

## References

[1] Verbeek JH , Rajamaki B , Ijaz S *et al*. Personal protective equipment for preventing highly infectious diseases due to exposure to contaminated body fluids in healthcare staff. Cochrane Database Syst. Rev. 2020; 4: CD011621. 32293717 10.1002/14651858.CD011621.pub4 PMC7158881

[2] Potter AW , Gonzalez JA , Xu X . Ebola response: modeling the risk of heat stress from personal protective clothing. PLoS One 2015; 10: e0143461. 26575389 10.1371/journal.pone.0143461 PMC4648492

[3] Bogdan A , Sudoł‐Szopińska I , Szopinski T . Assessment of textiles for use in operating theatres with respect to the thermal comfort of surgeons. Fibres Text. East. Eur. 2011; 19: 65–9.

[4] Coca A , Quinn T , Kim J‐H *et al*. Physiological evaluation of personal protective ensembles recommended for use in West Africa. Disaster Med. Public Health Prep. 2017; 11: 580–6. 28303774 10.1017/dmp.2017.13 PMC9901493

[5] Davey SL , Lee BJ , Robbins T *et al*. Heat stress and PPE during COVID‐19: impact on health care workers' performance, safety and well‐being in NHS settings. J. Hosp. Infect. 2021; 108: 185–8. 33301841 10.1016/j.jhin.2020.11.027 PMC7720696

[6] Ippolito M , Ramanan M , Bellina D *et al*. Personal protective equipment use by healthcare workers in intensive care unit during the early phase of COVID‐19 pandemic in Italy: a secondary analysis of the PPE‐SAFE survey. Ther. Adv. Infect. Dis. 2021; 8: 1–10. 10.1177/2049936121998562 PMC7922607 33717482

[7] Tabah A , Ramanan M , Laupland KB *et al*. Personal protective equipment and intensive care unit healthcare worker safety in the COVID‐19 era (PPE‐SAFE): an international survey. J. Crit. Care 2020; 59: 70–5. 32570052 10.1016/j.jcrc.2020.06.005 PMC7293450

[8] Messeri A , Bonafede M , Pietrafesa E *et al*. A web survey to evaluate the thermal stress associated with personal protective equipment among healthcare workers during the COVID‐19 pandemic in Italy. Int. J. Environ. Res. Public Health 2021; 18: 3861. 33917051 10.3390/ijerph18083861 PMC8067771

[9] Unoki T , Tamoto M , Ouchi A *et al*. Personal protective equipment use by healthcare workers in intensive care unit during the COVID‐19 pandemic in Japan: comparative analysis with the PPE‐SAFE survey. Acute Med. Surg. 2020; 7: e584. 33042559 10.1002/ams2.584 PMC7537292

[10] Bongers C , de Korte JQ , Zwartkruis M *et al*. Heat strain and use of heat mitigation strategies among COVID‐19 healthcare workers wearing personal protective equipment‐a retrospective study. Int. J. Environ. Res. Public Health 2022; 19: 1905. 35162925 10.3390/ijerph19031905 PMC8834922

[11] Lee J , Venugopal V , Latha PK *et al*. Heat stress and thermal perception amongst healthcare workers during the COVID‐19 pandemic in India and Singapore. Int. J. Environ. Res. Public Health 2020; 17: 8100. 33153079 10.3390/ijerph17218100 PMC7663197

[12] Australian Government . Guidance on the minimum recommendations for the use of personal protective equipment (PPE) in hospitals during the COVID‐19 outbreak (version 7). 2020.

[13] Hart SG . Nasa‐Task Load Index (NASA‐TLX); 20 years later. Proc. Hum. Factors Ergon. Soc. Annu. Meet. 2006; 50: 904–8.

[14] Hoonakker P , Carayon P , Gurses AP *et al*. Measuring workload of ICU nurses with a questionnaire survey: the NASA Task Load Index (TLX). IIE Trans. Healthc. Syst. Eng. 2011; 1: 131–43. 22773941 10.1080/19488300.2011.609524 PMC3388621

[15] International Organisation for Standardardisation . *Ergonomics of the thermal environment ‐ Assessment of the influence of the thermal environment using subjective judgement scales* (ISO 10551:2001). Geneva, 2001.

[16] Johnson RF , Merullo DJ . Subjective reports of heat illness. In: Marriot BM , ed. Nutritional Needs in Hot Environments: Applications for Military Personnel in Field Operations. Washington, DC: National Academic Press, 1993; 277–93. 25144014

[17] Sampson JB , Kobrick JL , Johnson RF . Measurement of subjective reactions to extreme environments: The environmental symptoms questionnaire. Mil. Psychol. 1994; 6: 215–33.

[18] Johnson EC , Ganio MS , Lee EC *et al*. Perceptual responses while wearing an American football uniform in the heat. J. Athl. Train. 2010; 45: 107–16. 20210614 10.4085/1062-6050-45.2.107 PMC2838462

[19] Fogarty A , Hunt A , Burdon C . Soldiers' perceived versus actual heat strain in a jungle environment. Extreme Physiol. Med. 2015; 4: A21.

[20] Hunt AP , Billing DC , Patterson MJ , Caldwell JN . Heat strain during military training activities: the dilemma of balancing force protection and operational capability. Temperature 2016; 3: 307–17. 10.1080/23328940.2016.1156801 PMC4965006 27857960

[21] Chappel SE , Aisbett B , Considine J , Ridgers ND . The accumulation of, and associations between, nurses' activity levels within their shift in the emergency department. Ergonomics 2020; 63: 1–10. 32757886 10.1080/00140139.2020.1807062

[22] Carter S , Field E , Oppermann E , Brearley M . The impact of perceived heat stress symptoms on work‐related tasks and social factors: a cross‐sectional survey of Australia's monsoonal north. Appl. Ergon. 2020; 82: 102918. 31473500 10.1016/j.apergo.2019.102918

[23] Jiang Q , Song S , Zhou J *et al*. The prevalence, characteristics, and prevention status of skin injury caused by personal protective equipment among medical staff in fighting COVID‐19: a multicenter, cross‐sectional study. Adv. Wound Care 2020; 9: 357–64. 10.1089/wound.2020.1212 PMC7307701 32320359

[24] Ong JJY , Bharatendu C , Goh Y *et al*. Headaches associated with personal protective equipment ‐ a cross‐sectional study among frontline healthcare workers during COVID‐19. Headache 2020; 60: 864–77. 32232837 10.1111/head.13811

[25] Yánez Benítez C , Güemes A , Aranda J *et al*. Impact of personal protective equipment on surgical performance during the COVID‐19 pandemic. World J. Surg. 2020; 44: 2842–7. 32564140 10.1007/s00268-020-05648-2 PMC7305697

