# Supplementary material for: Personal protective equipment for COVID‐19 among healthcare workers in an emergency department: An exploratory survey of workload, thermal discomfort and symptoms of heat strain
Source: Emerg Med Australas. 2022 Dec 20:10.1111/1742-6723.14152. Online ahead of print. doi: 10.1111/1742-6723.14152 (PMC9877975; doi:10.1111/1742-6723.14152)
Supplement: Supplementary file 1 — Appendix S1. NASA task load index. [file EMM-9999-0-s004.docx]

**Appendix S1: NASA Task load index**

The following questions ask about the workload that you experienced on your shift. Please slide the marker along each of the following six scales to the point that matches your experience. (Responses indicated on twenty-step scale, resulting in a score from 0 (low) to 100 (high))

**Mental demand:** How much mental activity was required during your shift (thinking, deciding, calculating, remembering, looking, searching, etc…)?

**Physical demand:** How much physical activity was required during your shift (e.g., pushing, pulling, turning, controlling, activating, etc.)?

**Temporal demand**: How much time pressure did you feel due to the rate or pace at which the tasks or task elements occurred?

**Effort:** How hard did you have to work (mentally and physically) to accomplish your level of performance?

**Performance:** How successful do you think you were?

**Frustration level:** How secure, gratified, content, relaxed and complacent did you feel during your shift (low frustration), compared to insecure, discouraged, irritated, stressed and annoyed (high frustration)?
