# Supplementary material for: Personal protective equipment for COVID‐19 among healthcare workers in an emergency department: An exploratory survey of workload, thermal discomfort and symptoms of heat strain
Source: Emerg Med Australas. 2022 Dec 20:10.1111/1742-6723.14152. Online ahead of print. doi: 10.1111/1742-6723.14152 (PMC9877975; doi:10.1111/1742-6723.14152)
Supplement: Supplementary file 2 — Appendix S2. Thermal discomfort. [file EMM-9999-0-s003.docx]

**Appendix S2: Thermal Discomfort**

The following questions ask about your thermal comfort during your shift. Please tick one box for each question that matches how you felt. Answer these questions with respect to how you felt while wearing PPE. If you did not wear PPE during your shift, answer these questions with respect to how you felt wearing your usual scrubs/uniform.

How did you feel during your shift?

- Hot
- Warm
- Slightly warm
- Neither hot nor cold
- Slightly cool
- Cool
- Cold

Did you find this feeling...

- Comfortable
- Slightly uncomfortable
- Uncomfortable
- Very uncomfortable
- Extremely uncomfortable

How would you have preferred to have felt?

- Much warmer
- Warmer
- A little warmer
- Neither warmer nor cooler
- A little cooler
- Cooler
- Much cooler

How sweaty did you feel?

- Not sweaty
- Slightly sweaty
- Sweaty
- Very sweaty

On a personal level, the thermal environment for me while wearing PPE (or usual scrubs/uniform if you did not wear PPE) was...

- Acceptable
- Unacceptable

In your opinion, was the thermal environment while wearing PPE (or usual scrubs/uniform if you did not wear PPE)...

- Perfectly tolerable
- Slightly difficult to tolerate
- Fairly difficult to tolerate
- Very difficult to tolerate
- Intolerable
