# Supplementary material for: Personal protective equipment for COVID‐19 among healthcare workers in an emergency department: An exploratory survey of workload, thermal discomfort and symptoms of heat strain
Source: Emerg Med Australas. 2022 Dec 20:10.1111/1742-6723.14152. Online ahead of print. doi: 10.1111/1742-6723.14152 (PMC9877975; doi:10.1111/1742-6723.14152)
Supplement: Supplementary file 3 — Appendix S3. Environmental Symptoms Questionnaire – subjective heat illness. [file EMM-9999-0-s001.docx]

**Appendix S3: Environmental Symptoms Questionnaire – Subjective Heat Illness**

The next 22 items list potential symptoms of heat illness. Please rate your experience of each symptom on a scale of Not at all, Slight, Somewhat, Moderate, Quite a bit, or Extreme.

| **SYMPTOM** | **NOT AT ALL** | **SLIGHT** | **SOMEWHAT** | **MODERATE** | **QUITE A BIT** | **EXTREME** |
| --- | --- | --- | --- | --- | --- | --- |
| I felt lightheaded | 0 | 1 | 2 | 3 | 4 | 5 |
| I had a headache | 0 | 1 | 2 | 3 | 4 | 5 |
| I felt dizzy | 0 | 1 | 2 | 3 | 4 | 5 |
| I felt faint | 0 | 1 | 2 | 3 | 4 | 5 |
| My coordination was off | 0 | 1 | 2 | 3 | 4 | 5 |
| I was short of breath | 0 | 1 | 2 | 3 | 4 | 5 |
| It was hard to breathe | 0 | 1 | 2 | 3 | 4 | 5 |
| My heart was beating fast | 0 | 1 | 2 | 3 | 4 | 5 |
| I had a muscle cramp | 0 | 1 | 2 | 3 | 4 | 5 |
| I had a stomach cramp | 0 | 1 | 2 | 3 | 4 | 5 |
| I felt weak | 0 | 1 | 2 | 3 | 4 | 5 |
| I felt constipated | 0 | 1 | 2 | 3 | 4 | 5 |
| I felt warm | 0 | 1 | 2 | 3 | 4 | 5 |
| I was sweating all over | 0 | 1 | 2 | 3 | 4 | 5 |
| Part of my body felt numb | 0 | 1 | 2 | 3 | 4 | 5 |
| My vision was blurry | 0 | 1 | 2 | 3 | 4 | 5 |
| I lost my appetite | 0 | 1 | 2 | 3 | 4 | 5 |
| I felt sick | 0 | 1 | 2 | 3 | 4 | 5 |
| I was thirsty | 0 | 1 | 2 | 3 | 4 | 5 |
| I felt tired | 0 | 1 | 2 | 3 | 4 | 5 |
| I felt irritable | 0 | 1 | 2 | 3 | 4 | 5 |
| I felt restless | 0 | 1 | 2 | 3 | 4 | 5 |
