# Supplementary material for: Personal protective equipment for COVID‐19 among healthcare workers in an emergency department: An exploratory survey of workload, thermal discomfort and symptoms of heat strain
Source: Emerg Med Australas. 2022 Dec 20:10.1111/1742-6723.14152. Online ahead of print. doi: 10.1111/1742-6723.14152 (PMC9877975; doi:10.1111/1742-6723.14152)
Supplement: Supplementary file 4 — Table S1. Individual comments about wearing PPE. [file EMM-9999-0-s002.docx]

**Table S1: Individual comments about wearing PPE**

| Heat-related issues | |
| --- | --- |
|  | - Mostly the heat and sweating from full gowns leaving me feeling dehydrated and fatigued. |
|  | - When in full PPE, very hot, thirsty, sweaty, lightheaded, headache and often faint. Wearing a mask all shift you feel thirsty, slightly sweaty and headache. |
|  | - Today is a cool day so not really causing much thermal discomfort. |
|  | - I sweat a bit during everyday life, wearing PPE I always sweat quite a bit that it drips off every area, always dizzy and lightheaded. |
|  |  |
| Other PPE issues | |
|  | - Itch from fibres. |
|  | - Main issue was pressure of mask over nasal bone. With normal face mask wearing glasses is unbearable. |
|  | - The mask fogs my glasses. Requiring more cleaning. Thanks. |
|  | - Quality of PPE; in particular face masks is below standard. |
|  | - Main issue is discomfort of nose and ears from wearing masks. |
|  |  |
| Work Practices | |
|  | - I have worn PPE for many years in ICU for up to 12 hrs a day so I am used to it. What is the issue in ED is the time it takes to don and doff it. Also, layout of department makes that difficult, not much to do about that as you cannot change the footprint of the unit. |
|  | - The slight warmth I felt was limited to my face. Irritation related to ear pain from my mask. The greatest discomfort was in the inability to have a drink (water or coffee) without leaving the floor, which is usually inconvenient for patient care. Having a coffee while I work is a bright spot in my day that is not possible with PPE. |
|  | - When wearing a duckbill or n95 mask for a 12-hour shift, I inevitability end up drinking less water which makes me feel awful by the end of the day. I also get a headache from the pressure of the mask and get a sore red nose and cheeks with indents in my skin from the mask. |
|  | - I believe staff wearing N95s should have frequent breaks to allow for adequate hydration. |
|  | - It affects me if my physical activity goes up- e.g., climbing stairs. It feels more difficult to get a relieving deep breath and clear CO2 build up. I have had to go outside of breaks to get a sense of breathing freely. When I’m more physically active I have noticed more sweating and need to remove cardigan to cool down. I had not needed to go outside or decrease clothing prior to masks |
|  | - PPE is part of life when you work in health care, people need to stop being precious and just suck it up, this is life. |
|  |  |
| Effects on Performance | |
|  | - Decreases efficiency significantly. |
